# Increase in cell motility by carbon ion irradiation via the Rho signaling pathway and its inhibition by the ROCK inhibitor Y-27632 in lung adenocarcinoma A549 cells

**DOI:** 10.1093/jrr/rru002

**Published:** 2014-03-21

**Authors:** Kazutoshi Murata, Shin-ei Noda, Takahiro Oike, Akihisa Takahashi, Yukari Yoshida, Yoshiyuki Suzuki, Tatsuya Ohno, Tomoo Funayama, Yasuhiko Kobayashi, Takeo Takahashi, Takashi Nakano

**Affiliations:** 1 Department of Radiation Oncology, Gunma University Graduate School of Medicine, 3-39-22, Showa-machi, Maebashi, Gunma 371-8511, Japan; 2 Advanced Scientific Research Leaders Development Unit, Gunma University, 3-39-22, Showa-machi, Maebashi, Gunma 371-8511, Japan; 3 Gunma University Heavy Ion Medical Center, 3-39-22, Showa-machi, Maebashi, Gunma 371-8511, Japan; 4 Microbeam Radiation Biology Group, Japan Atomic Energy Agency, Watanuki 1233, Takasaki, Gunma 370-1292, Japan; 5 Department of Radiation Oncology, Saitama Medical Center, Saitama Medical University, 1981, Kamoda, Kawagoe, Saitama 350-8550, Japan

**Keywords:** non-small cell lung carcinoma (NSCLC), cell motility, carbon ion (C-ion) irradiation, *ras* homolog gene family member (Rho), Rho-associated coiled-coil-forming protein kinase (ROCK)

## Abstract

This study aimed to investigate the effect of carbon ion (C-ion) irradiation on cell motility through the ras homolog gene family member (Rho) signaling pathway in the human lung adenocarcinoma cell line A549. Cell motility was assessed by a wound-healing assay, and the formation of cell protrusions was evaluated by F-actin staining. Cell viability was examined by the WST-1 assay. The expression of myosin light chain 2 (MLC2) and the phosphorylation of MLC2 at Ser19 (P-MLC2-S19) were analyzed by Western blot. At 48 h after irradiation, the wound-healing assay demonstrated that migration was significantly greater in cells irradiated with C-ion (2 or 8 Gy) than in unirradiated cells. Similarly, F-actin staining showed that the formation of protrusions was significantly increased in cells irradiated with C-ion (2 or 8 Gy) compared with unirradiated cells. The observed increase in cell motility due to C-ion irradiation was similar to that observed due to X-ray irradiation. Western-blot analysis showed that C-ion irradiation (8 Gy) increased P-MLC2-S19 expression compared with in unirradiated controls, while total MLC2 expression was unchanged. Exposure to a non-toxic concentration of Y-27632, a specific inhibitor of Rho-associated coiled-coil-forming protein kinase (ROCK), reduced the expression of P-MLC2-S19 after C-ion irradiation (8 Gy), resulting in a significant reduction in migration. These data suggest that C-ion irradiation increases cell motility in A549 cells via the Rho signaling pathway and that ROCK inhibition reduces that effect.

## INTRODUCTION

Non-small cell lung carcinoma (NSCLC) is one of the most lethal types of cancer, showing resistance to conventional radiation therapy (RT) [1]. Carbon ion (C-ion) RT is considered to be a promising treatment strategy for early-stage NSCLC because (i) C-ion RT is superior to conventional X-ray RT in dose distributions, with higher concentrations in tumors and superior normal tissue sparing that enables dose escalations, and (ii) a C-ion beam has a higher relative biological effectiveness (RBE) than that of X-rays. In fact, a previous study demonstrated favorable local control achieved by C-ion RT in patients with early-stage NSCLC [2]; however, another previous study documented cases with marginal recurrence after C-ion RT, which is an issue of great importance [3]. Marginal recurrence in C-ion RT may be due, in part, to insufficient dose delivery with a steep dose fall-off and susceptibility to set-up error and organ motion at the margin of tumors. Another possible cause for marginal recurrence is increased cancer-cell motility after irradiation. Several *in vitro* studies demonstrated that X-ray irradiation increased cell motility in cancer cells of various origins, including lung [4–6]. These reports indicate the possibility that cancer cells receiving X-ray irradiation may move outside of the radiation fields in clinical settings.

The increase in cancer-cell motility due to X-ray irradiation is achieved through the *ras* homolog gene family member (Rho) signaling pathway [5, 7]. In the Rho signaling pathway, Rho-associated coiled-coil-forming protein kinase (ROCK) is considered to act as an effector downstream of Rho [8]. ROCK directly and indirectly phosphorylates myosin light chain 2 (MLC2) at Ser19 (P-MLC2-S19), thereby increasing the contractility of actomyosin, which forms stress fibers and cell protrusions, resulting in cell migration. However, the effect of C-ion irradiation on motility in NSCLC cells and the underlying mechanisms have not been fully elucidated. Therefore, the present study investigated motility in A549 lung adenocarcinoma cells exposed to C-ion irradiation *in vitro*. The involvement of the Rho signaling pathway in radiation-induced motility was assessed using Y-27632, a specific inhibitor of ROCK [9].

## MATERIALS AND METHODS

### Cells and materials

A549 cells were purchased from the Cell Resources Center for Biomedical Research in Tohoku University (Sendai, Japan). The cells were cultured in RPMI-1640 (Invitrogen, CA, USA) supplemented with 10% fetal bovine serum (ICN Biomedicals, OH, USA) at 37°C with 5% CO_2_. Y-27632, a specific inhibitor of ROCK, was purchased from Merck (Darmstadt, Germany).

### Carbon ion and X-ray irradiation

C-ion irradiation was performed at the Takasaki Ion Accelerators for Advanced Radiation Application in the Japan Atomic Energy Agency (Gunma, Japan) or Gunma Heavy Ion Medical Center (Gunma, Japan). C-ion beams with a linear energy transfer of 108 keV/μm were used. The RBE of the C-ion irradiation in A549 cells at a 10% surviving fraction was calculated as 3.9 by a clonogenic survival assay (Supplementary Fig. 1).

**Fig. 1.**
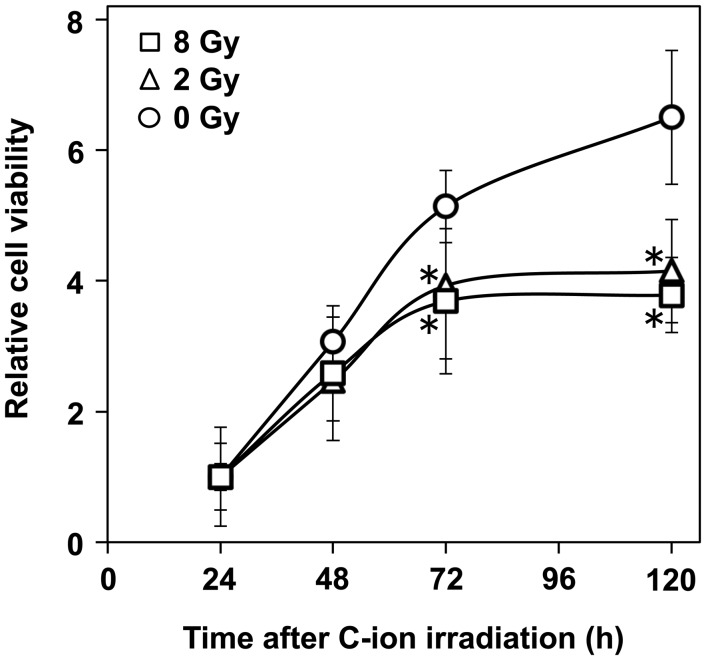
The effect of carbon (C-ion) irradiation on the viability of A549 cells assessed by WST-1 assay. An asterisk indicates significant difference compared with the unirradiated control.

### WST-1 assay

To assess the viability of A549 cells, a WST-1 assay (Roche, Mannheim, Germany) was performed according to the manufacturer's instructions. A549 cells were seeded on 96-well plates (1.0 × 10^4^ cells/well) and incubated at 37°C for 12 h. The cells received C-ion irradiation (0, 2 or 8 Gy) or continuous exposure to Y-27632 (0.01, 0.1, 1, 10 or 50 µM). After incubating for 24, 48, 72 or 120 h, 20 µl of WST-1 reagent was applied to each well. After incubating for an additional 3 h at 37°C, absorption of 450 nm light was measured using a Multiskan FC (Thermo, Helsinki, Finland).

### Wound-healing assay

The motility of A549 cells was assessed by wound-healing assay, as previously described [7]. Briefly, A549 cells were cultured on 35-mm culture dishes to confluence. The monolayers were wounded by scratching with sterile pipette tips, rinsed once with culture medium, and then exposed to fresh culture medium. The cells were then subjected to C-ion irradiation (2 or 8 Gy) and incubated at 37°C for 2, 6, 12, 24 or 48 h. Phase-contrast images of the wounds were obtained at a × 20 magnification using a Real Surface View Microscope VE-7800 (Keyence, Osaka, Japan). For each wound, the distance between the nuclei of the cells located on the two edges of a wound was measured at 30 points in the vertical direction to the axis of the wound; the average value was defined as the width of the wound. The distance the cells migrated was calculated by subtracting the width of the wound measured at a given timepoint from that obtained 2 h after irradiation. To assess the effect of Y-27632, the cells were continuously exposed to 30 µM Y-27632 from 18 h before wounding to image acquisition.

### F-actin staining

A549 cells seeded on coverslips were cultured for 18 h at 37°C to achieve 50% confluence, and then subjected (or not) to X-ray (8 Gy) or C-ion irradiation (2 or 8 Gy). After culturing for a further 24 h, the cells were fixed with 3.7% paraformaldehyde for 10 min at room temperature, and permeabilized with 0.2% Triton X-100 for 5 min. The specimens were incubated with the actin stain 488 phalloidin (Cytoskeleton Inc., CO, USA) for 30 min at 37°C according to the manufacturer's instruction, and counter-stained with DAPI for 5 min. To evaluate the formation of cell protrusions, the cells were imaged using a Real Surface View Microscope VE-7800 (Keyence) at a ×20 magnification. Five independent cell counts of at least 100 cells each were performed for each set of experimental conditions. The cells were counted by two researchers (K.M. and T.O.), blind to the experimental conditions, and evaluated for the formation of cell protrusions.

### Western-blot analysis

A549 cells were cultured in the presence or absence of Y-27632 (30 µM) for 18 h and subjected (or not) to C-ion irradiation (2 or 8 Gy). After incubating for a further 0.5, 2 or 24 h at 37°C, the cells were harvested and lysed with cell lysis buffer (Millipore, MA, USA) containing phosphatase inhibitor cocktails 1 and 2 (Sigma-Aldrich, MO, USA) and protease inhibitor cocktail 3 (Calbiochem, Darmstadt, Germany). The protein lysates (50 µg) were subjected to electrophoresis on 15% mini-PROTEAN TGX precast gels (Bio-Rad, CA, USA) and transferred to nitrocellulose membranes (Bio-Rad). The membranes were incubated at room temperature for 1 h with PBST supplemented with 5% skimmed milk and exposed to primary and secondary antibodies. Primary antibodies specific for the following proteins were used: MLC2 (Cell Signaling Technology, MA, USA), P-MLC2-S19 (Cell Signaling Technology) and β-actin (Chemicon International, CA, USA). A polyclonal goat anti-mouse immunoglobulin (DAKO, CA, USA) was used as a secondary antibody. The Western blots were visualized using ECL chemiluminescent reagents (Bio-Rad) and ChemiStage CC16 mini (Wealtec, NV, USA).

### Statistics

Significant differences were analyzed by an unpaired two-tailed Student's t-test. *P* < 0.05 was considered significant. Results are expressed as the mean ± standard deviation (SD) of values obtained from at least three independent experiments. The error bars in the figures represent SDs.

## RESULTS

### Carbon ion irradiation increased cell motility in A549 cells

First we examined the effect of C-ion irradiation on the motility of A549 cells. Prior to the assessment of cell motility, the viability of C-ion-irradiated cells was assessed because differences in viability among cells receiving different doses of C-ion irradiation might affect the motility assessment. The WST-1 assay showed that the viability of the unirradiated cells increased from 24 to 120 h (Fig. 1). Meanwhile, C-ion irradiation (2 and 8 Gy) significantly reduced the cell viability 72 and 120 h after the irradiation (2 Gy, *P =* 0.0026 and 0.000 70; 8 Gy, *P =* 0.013 and 0.0027, respectively); however, 48 h after receiving C-ion irradiation, there were no significant differences in viability between the irradiated and unirradiated cells (0 Gy vs 2 Gy, *P =* 0.22; 0 Gy vs 8 Gy, *P =* 0.49). As a result of these data, the assessment of cell motility was performed within 48 h after irradiation, a period when C-ion irradiation did not significantly affect cell viability.

**Fig. 2.**
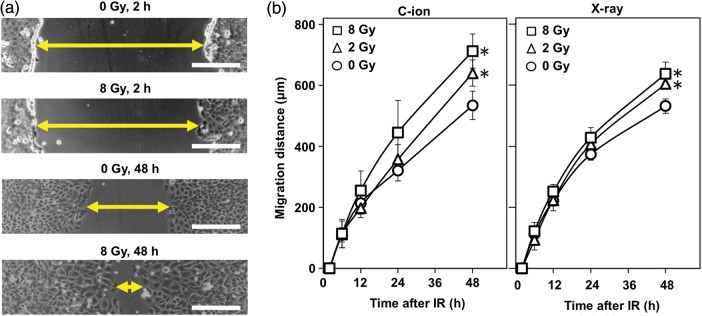
The effect of carbon ion (C-ion) and X-ray irradiation on cell motility in A549 cells assessed by a wound-healing assay. **(a)** Representative micrographs of the wounds observed 2 and 48 h after receiving C-ion irradiation. The arrow indicates the width of a wound. Scale bar, 200 µm. **(b)** Distances the cells migrated. Left panel, cells receiving C-ion irradiation; right panel, cells receiving X-ray irradiation. An asterisk indicates significant difference compared with the corresponding unirradiated control.

The effect of C-ion irradiation on cell motility was assessed by wound-healing assay (Fig. 2a and b). At 48 h after receiving C-ion irradiation for 0, 2 or 8 Gy, the distance the cells migrated was 534 ± 47, 640 ± 43 and 712 ± 56 µm, respectively. The migration distance was significantly greater in the cells irradiated with 2 and 8 Gy than in the unirradiated cells (*P =* 0.045 and 0.013, respectively). It appeared that there was a dose-dependence in the increase in cell motility.

Meanwhile, the wound-healing assay showed that X-ray irradiation also increased the motility of A549 cells (Fig. 2b). At 48 h after X-ray irradiation (2 and 8 Gy), when cell viability was not significantly affected (data not shown), the cells receiving 0, 2 and 8 Gy migrated 531.52 ± 23.67, 604.12 ± 16.55 and 637.58 ± 38.35 µm, respectively. The migration distance was significantly greater in the cells receiving X-ray irradiation (2 and 8 Gy) than in the unirradiated cells (*P =* 0.012 and 0.015, respectively).

**Fig. 3.**
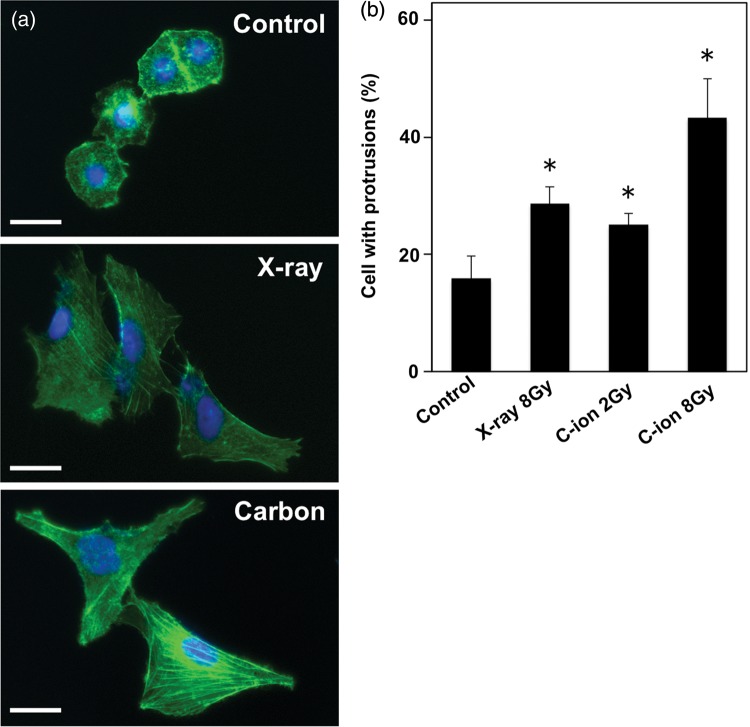
The effect of carbon ion (C-ion) and X-ray irradiation on the formation of cell protrusions in A549 cells assessed by F-actin staining. **(a)** Representative micrograph of non-treated control cells (upper panel), cells irradiated with X-rays for 8 Gy (middle panel), and those irradiated with C-ion for 8 Gy (lower panel) taken 24 h after irradiation at a ×40 magnification. F-actin and nuclei are shown in green (Alexa Fluor 488 phaloidin) and blue (DAPI), respectively. Scale bar, 30 µm. **(b)** Numbers of cells forming protrusions are shown. An asterisk indicates significant difference compared with the unirradiated control.

To further investigate the effect of C-ion and X-ray irradiation on cell motility, we evaluated the formation of cell protrusions, another marker of motility [10], by F-actin staining. Interestingly, irradiation with both C-ion (2 and 8 Gy) and X-rays (8 Gy) significantly increased the formation of cell protrusions 24 h after irradiation compared with unirradiated controls (C-ion, *P =* 0.0026 and 0.000 039 for 2 and 8 Gy, respectively; X-rays, *P* = 0.000 28 for 8 Gy) (Fig. 3). Taken together, these data suggest that C-ion irradiation increases cell motility in A549 cells in a similar manner to X-rays.

### ROCK inhibition by Y-27632 suppressed the increase in motility in A549 cells after carbon ion irradiation

**Fig. 4.**
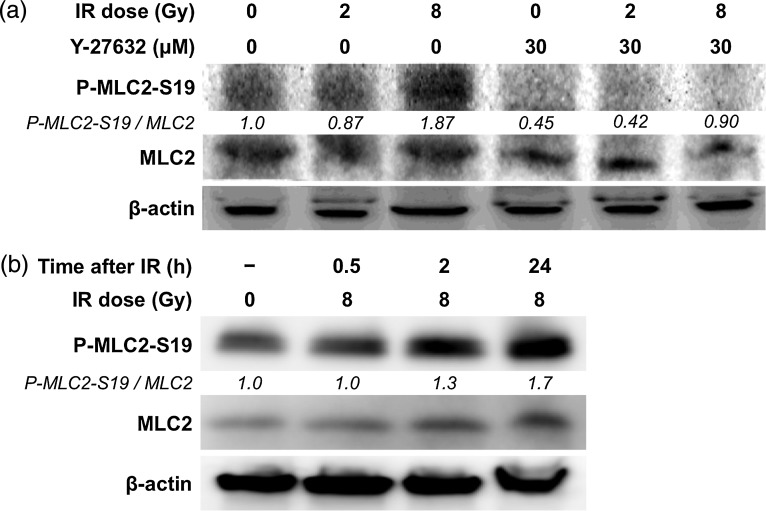
The effects of carbon ion (C-ion) irradiation and/or Y-27632 on the phosphorylation of myosin light chain 2 (MLC2) in A549 cells assessed by Western blot. The ratio of the expression level of phosphorylated MLC2 at Ser19 (P-MLC2-S19) to that of MLC2 is shown. The ratios were normalized with respect to the non-treated control. β-actin is shown as a loading control. IR = irradiation. **(a)** Expression of MLC2 and P-MLC2-S19 in cells exposed to C-ion IR (2 or 8 Gy) and/or Y-27632 (30 µM) 24 h after irradiation. **(b)** Expression of MLC2 and P-MLC2-S19 in cells irradiated with C-ion (8 Gy) 0.5, 2 or 24 h after irradiation.

**Fig. 5.**
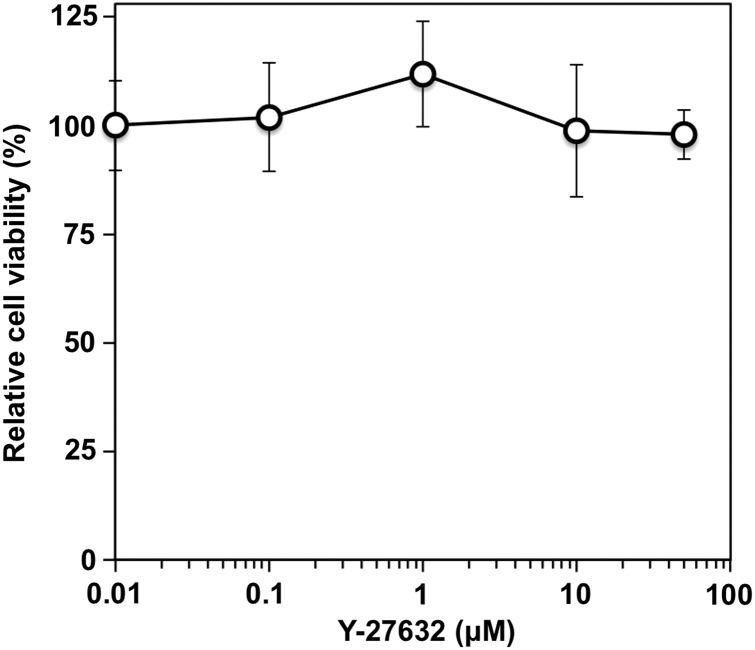
The effect of Y-27632 on the viability of A549 cells. The cells were exposed to Y-27632 for 48 h and subjected to a WST-1 assay. The viability of cells were normalized with respect to that of cells exposed to 0.01 µM Y-27632.

**Fig. 6.**
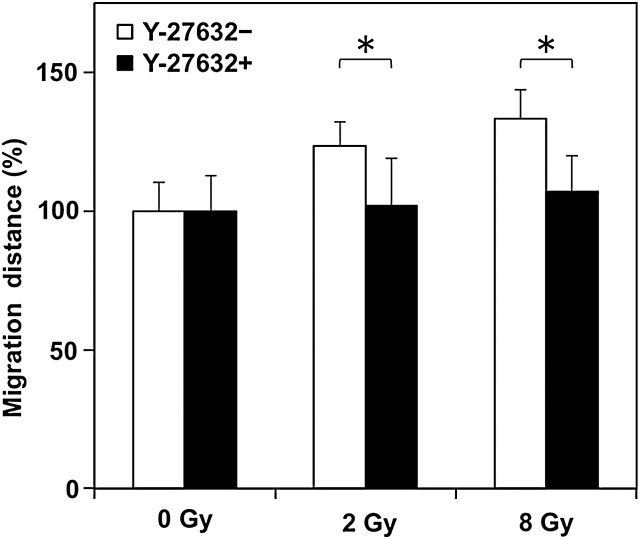
The effect of Y-27632 on motility in A549 cells after C-ion irradiation assessed by a wound-healing assay. The cells were exposed to Y-27632 (30 µM) for 18 h and subjected to a wound-healing assay as in Fig. 2. The cells were kept exposed to Y-27632 until the time of assessment. For each of the samples treated with or without Y-27632, the migration distances were compared with those of unirradiated controls. The asterisk indicates significant difference.

The Rho signaling pathway is involved in the increase in cell motility after X-ray irradiation in A549 cells [7]. Therefore, the involvement of the Rho signaling pathway in the increase in cell motility after C-ion irradiation in A549 cells was investigated by Western blot. At 24 h after C-ion irradiation, increased P-MLC2-S19 expression was observed in cells irradiated with 8 Gy compared with unirradiated controls (Fig. 4a). Interestingly, the phosphorylation level of MLC2 increased in a time-dependent manner (Fig. 4b). These results indicate that activation of the Rho signaling pathway may contribute to the increase in cell motility in A549 cells receiving C-ion irradiation. Therefore, we investigated whether the inhibition of ROCK suppressed the increase in cell motility by C-ion irradiation using Y-27632, which is a specific inhibitor of ROCK. A WST-1 assay showed that there was no significant difference in the viability of the cells exposed to Y-27632 at concentrations ranging from 0.01 to 50 µM for 48 h (Fig. 5). From these results, 30 µM, a concentration at which Y-27632 does not affect cell viability, was used in subsequent analyses. Western-blot analysis showed that the expression of P-MLC2-S19 was reduced in all the samples exposed to Y-27632 (Fig. 4a). Of note, Y-27632 reduced the expression of P-MLC2-S19 in cells receiving C-ion (8 Gy) to a level similar to that observed in non-treated controls, indicating that Y-27632 suppressed signal transduction through the Rho/ROCK/MLC2 pathway activated after C-ion irradiation. Wound-healing assays revealed that Y-27632 significantly reduced the migration distance of the irradiated cells (*P =* 0.049 and 0.039 for 2 and 8 Gy, respectively) (Fig. 6). Taken together, these data indicate that inhibition of ROCK reduces the increase in cell motility after C-ion irradiation in A549 cells.

## DISCUSSION

In the present study, wound-healing assays and F-actin staining showed that C-ion irradiation increased cell motility in A549 cells. It seems that the increase in cancer-cell motility after irradiation is not a characteristic of C-ion beams, but rather a phenomenon similar to what is observed after X-ray irradiation. We also found that the Rho/ROCK/MLC2 signaling pathway, which can be targeted by the ROCK inhibitor Y-27632, was involved in the increase in cell motility in A549 cells after C-ion irradiation, suggesting that ROCK inhibition may reduce the migration of the irradiated cancer cells when used in combination with C-ion RT.

Several *in vitro* studies investigated the effect of C-ion irradiation on the migration of cancer cells [11, 12]. In these, C-ion irradiation suppressed the migration of cancer cells (U87 glioma cells and HCT116 colon carcinoma cells by Goetze *et al*.; EBC-1 lung squamous cell carcinoma cells and A549 cells by Akino *et al*.). These results are in contrast to ours, possibly due to the difference in the assay system employed. Our study used a wound-healing assay and evaluated the migration of cells in a horizontal direction on culture dishes. Meanwhile, Goetze *et al*. [11] and Akino *et al*. [12] used a Boyden chamber assay in which the numbers of cells that pass through a filter with narrow pores in a vertical direction are counted. Moreover, the studies by Goetze *et al*. and Akino *et al*. employed filters precoated with collagens. Cancer cells utilize proteolytic enzymes such as matrix metalloproteinases to degrade the extracellular matrix, including collagens, and generate a path for moving through it [13]. Therefore, in the studies by Goetze *et al*. and Akino *et al*., the effect of C-ion irradiation on the activity of proteolytic enzymes may be reflected in the results. Further investigation is required to elucidate the effect of C-ion irradiation on cell motility, proteolytic enzyme activity and other mechanisms involved in cancer invasion and metastasis.

The results of the present study indicate that cell motility in A549 cells is increased by C-ion irradiation with 2 and 8 Gy, calculated as 7.8 and 31.2 GyRBE, respectively. The irradiation dose per fraction used in C-ion RT for early-stage NSCLC at Gunma University Heavy Ion Medical Center falls within this range (i.e. 13.2–15.0 GyRBE/fraction; a total of 52.8–60.0 GyRBE is delivered in four fractions in one week). Therefore, although it is crude to directly extrapolate *in vitro* data into the clinical setting, the results of the wound-healing assay suggest a potential clinical benefit. In fact, the migration distance of the irradiated cells scored in the wound-healing assay corresponded to 0.53–0.71 mm per day. These data may be informative in defining clinical target volumes in actual treatment planning. To further explore this issue, the effect of the fractionated C-ion irradiation on cancer-cell motility should be examined.

The Rho signaling pathway involving in the cell motility has many downstream effectors. Among them, in the present study, we selected the phosphorylation of MLC2 at Ser19 as an indicator of the activation of Rho signaling because it is well known that the Ser19 of MLC2 is specifically phosphorylated via ROCK [14], and that the resulting P-MLC2-S19 plays a pivotal role in cell motility through its interaction with actin–myosin [15]. There is a clinically available (intravenously administered) agent with ROCK inhibitory activity named fasudil (Daiichi Chemical and Pharmacological Company, Ibaragi, Japan), which is approved in Japan for the prevention of cerebral vasospasm in patients with subarachnoid hemorrhage [16]. In this regard, if the involvement of the Rho/ROCK/MLC2 cascade in the increased cancer-cell motility after C-ion irradiation is proven, the finding can be directly applied in the clinic with fasudil treatment combined with C-ion RT for the prevention of cancer-cell migration after C-ion RT. However, fasudil is known to interact with multiple molecules such as free intracellular calcium ions and protein kinases A, G and C (16). Thus, in the present study, in order to clearly evaluate the effect of ROCK inhibition on cell motility after C-ion irradiation, the inhibitor Y-27632, which is specific for ROCK [17], was used instead of fasudil, even though Y-27632 is not suitable for human use. As a result, ROCK inhibition by Y-27632 resulted in the reduced motility in A549 cells irradiated with C-ion of 2 and 8 Gy by 20% and 25%, respectively (Fig. 6). These data indicate the potential of clinical ROCK inhibition for the suppression of migration of cancer cells during and after C-ion RT. Taken together, fasudil combined with C-ion RT is an attractive method for improving local disease control that warrants investigation in the clinic.

The present study has several limitations. In a wound-healing assay, only the motility of cells at the edge of scratched wounds can be evaluated. In contrast, in F-actin staining, cells seeded on coverslips at 50% confluence showed increased cell protrusions, indicating that C-ion irradiation increased cell motility not only in cells living on the edge of tumors, but also in those living at the center of a cluster of cancer cells. Nevertheless, these results are not enough to completely understand the spatio-temporal effect of C-ion irradiation on cancer-cell motility. The present study also investigated the effect of C-ion irradiation on cancer-cell motility within 48 h after irradiation, when C-ion irradiation did not significantly affect cell viability, in order to focus on motility; however, in the clinical setting the situation can be more complicated because the repopulation of the irradiated cancer cells may affect their motility. To clarify these issues, analyses using 3D tumor models such as *in vitro* spheroids or *in vivo* mouse xenograft tumor models should be performed in the future. Furthermore, the mechanism underlying the continuous phosphorylation of MLC2-S19 after C-ion irradiation is largely unknown and warrants further investigation.

## CONCLUSION

In conclusion, the present study showed that C-ion irradiation increased cell motility in A549 cells via the Rho/ROCK/MLC2 signaling pathway, and that inhibition of ROCK reduced the increase in cell motility in irradiated cells. Further investigation will elucidate the effect of C-ion irradiation on migration, invasion and metastasis, and the underlying mechanism, which will help to develop efficient treatment strategies in C-ion RT.

## SUPPLEMENTARY DATA

Supplementary data is available at the *Journal of Radiation Research* online.

## FUNDING

This work was supported by Grant-in-Aid for Young Scientists (B) from the Japan Society for the Promotion of Science Grant Number 25861071.

## Supplementary Material

Supplementary Data
