# Supplementary material for: Increase in cell motility by carbon ion irradiation via the Rho signaling pathway and its inhibition by the ROCK inhibitor Y-27632 in lung adenocarcinoma A549 cells
Source: J Radiat Res. 2014 Mar 21;55(4):658–64. doi: 10.1093/jrr/rru002 (PMC4099995; doi:10.1093/jrr/rru002)
Supplement: Supplementary Data [file supp_55_4_658__index.html]

Increase in cell motility by carbon ion irradiation via the Rho signaling pathway and its inhibition by the ROCK inhibitor Y-27632 in lung adenocarcinoma A549 cells — Increase in cell motility by carbon ion irradiation via the Rho signaling pathway and its inhibition by the ROCK inhibitor Y-27632 in lung adenocarcinoma A549 cells — Supplementary Data 

# Increase in cell motility by carbon ion irradiation via the Rho signaling pathway and its inhibition by the ROCK inhibitor Y-27632 in lung adenocarcinoma A549 cells

## Supplementary Data

Supplementary Data

**Files in this Data Supplement:**

- Supplementary Figure 1 - tif file
