# Supplementary figures and images for: Increase in cell motility by carbon ion irradiation via the Rho signaling pathway and its inhibition by the ROCK inhibitor Y-27632 in lung adenocarcinoma A549 cells
Source: J Radiat Res. 2014 Mar 21;55(4):658–64. doi: 10.1093/jrr/rru002 (PMC4099995; doi:10.1093/jrr/rru002)

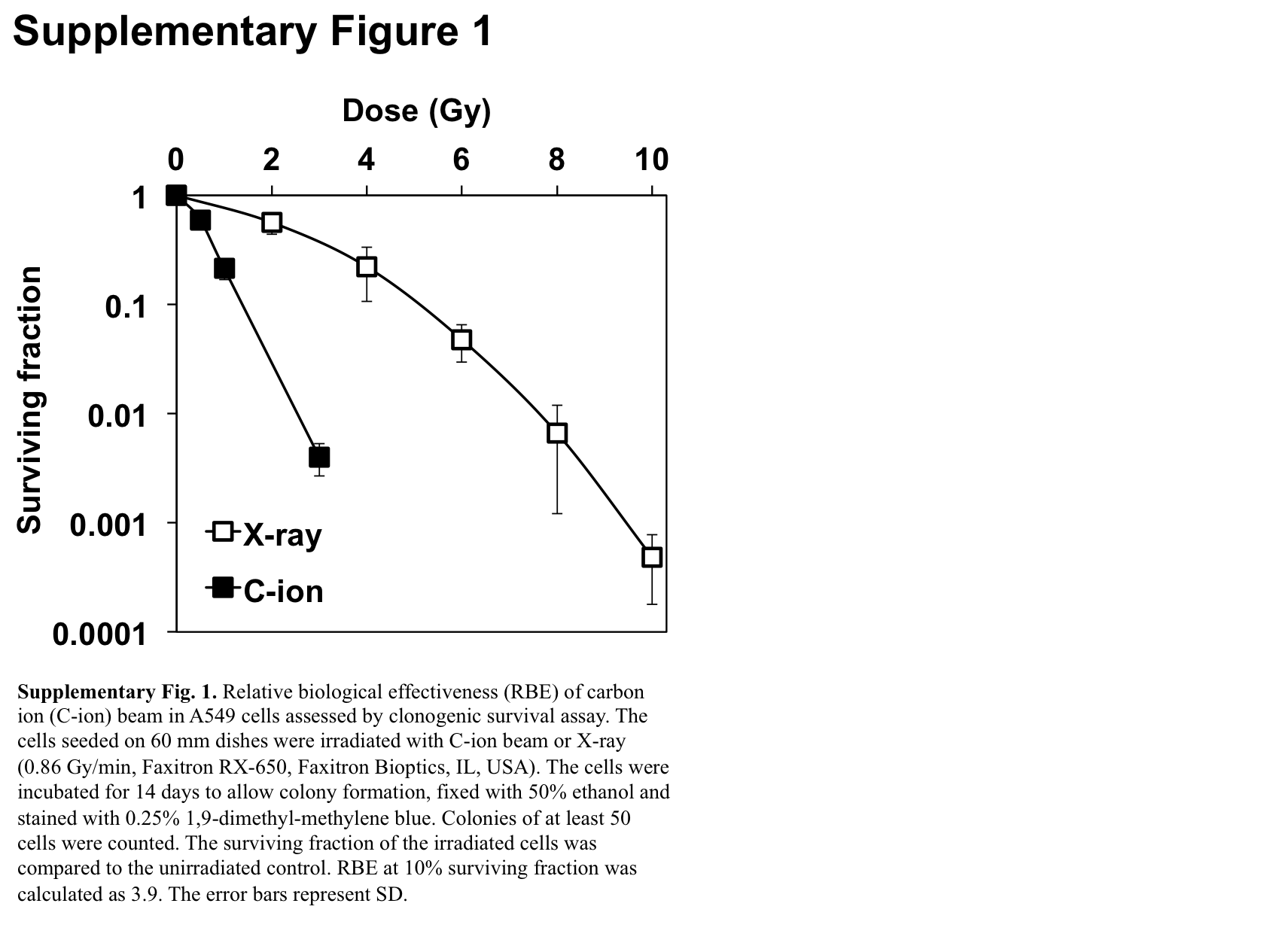

Supplement: Supplementary Data [file supp_rru002_rru002supp_fig1.tif]
